# Diagnostic Performance of Neck Circumference and Cut-off Values for Identifying Overweight and Obese Pakistani Children: A Receiver Operating Characteristic Analysis

**DOI:** 10.4274/jcrpe.galenos.2020.2019.0212

**Published:** 2020-11-25

**Authors:** Muhammad Asif, Muhammad Aslam, Justyna Wyszyńska, Saima Altaf, Shakeel Ahmad

**Affiliations:** 1 Govt. Degree College, Qadir Pur Raan, Department of Statistics, Multan, Pakistan; 2 Bahauddin Zakariya University, Department of Statistics, Multan, Pakistan; 3 Medical College of Rzeszów University, Rzeszów, Poland

**Keywords:** Body mass index, LMS method, neck circumference, obesity, receiver operating characteristic curve

## Abstract

**Objective:**

Neck circumference (NC) is considered to be an alternative screening method for obesity. The aims were: (1) to examine the correlation between body mass index (BMI) and NC; and (2) to determine diagnostic performance including the best cut-off values of NC for identification of overweight and obese Pakistani children.

**Methods:**

The study sample was 7,921 children, aged 5-14 years, by cross-sectional survey carried-out in four major cities of Pakistan. Receiver operating characteristic analysis was used to investigate the diagnostics performance of NC and to determine the optimal cut-off points for identifying children with overweight and obesity.

**Results:**

The mean of each anthropometric variable (i.e., height, weight, BMI and NC) increased with age in both sexes. In the whole sample, NC had a strong positive correlation (r=0.61, p<0.01) with BMI. NC optimal cut-off points for identifying overweight and obesity in Pakistani boys ranged between 25.00 to 30.35 cm and the corresponding values for the girls were 24.00 to 31.62 cm. In the prepubertal period, NC cut-off points indicative overweight, in both boys and girls were 26.36 cm and 25.27 cm, respectively; the corresponding values for obesity were 26.78 cm and 25.02 cm. During puberty, the cut-off values for overweight and obesity respectively were 28.32 cm and 28.57 cm in boys and 28.70 cm and 28.82 cm in girls.

**Conclusion:**

NC may be used as a simple and widely applicable measure for identification of overweight and obesity with reasonable accuracy in Pakistani children.

**What is already known on this topic?**

Childhood obesity is a growing problem in Pakistan. Therefore there is a need to identify a quick and simple tool for screening obesity. Neck circumference (NC) may be a valuable tool for screening individuals with overweight/obesity.

**What this study adds?**

This is the first study evaluating the correlation between NC and body mass index among Pakistani children. The optimal cut-off values of NC for identification of overweight and obesity were identified in pre-pubertal and pubertal boys and girls using receiver operating characteristic analysis.

## Introduction

In recent decades, obesity has become an increasing global public health issue (1,2,3,4). Children and adolescents are the worst affected group with an estimated 10% of the world’s school children being overweight and one quarter of these being obese (4,5). In developing countries including Pakistan, childhood obesity is also growing at a fast pace. Different studies (6,7,8,9) in various settings show that the prevalence of overweight and obesity in Pakistani children ranges from 8% to 19.3% and 6% to 7.5%, respectively.

To measure obesity prevalence in children and adults, there are various anthropometric measures. However, epidemiological researchers usually use the internationally recognized and established measure body mass index (BMI), which is calculated by taking an individual’s weight in kilograms (kg) and dividing by height in meters squared (2). Despite the popularity of BMI and ease of use, it is becoming increasingly clear that it is not a good measure for regional adiposity, especially upper body fat distribution of an individual (10).

Currently, neck circumference (NC) is an alternative screening method, proposed as a potential proxy for BMI (11,12). Measurement of NC is an easy, quick and inexpensive method and various investigators have attempted to use it for screening of overweight and obese children (13,14). Studies with different pediatric samples showed that NC performed well as an index of high BMI in young children and adolescents (13,15,16).

However in Pakistan, there is a scarcity of data about the use of NC as an indicator of overweight and obesity in children. Only one investigator (17) has attempted to use NC to screen for high BMI among young adults aged 18-20 years. Given this gap in the evidence base the present study was undertaken with the following objectives: i) to evaluate the correlation between NC and BMI in children and ii) to determine diagnostic performance and the best NC cut-off values for identification of overweight and obese Pakistani children.

## Methods

This was a school-based, cross-sectional study and was conducted between March and June, 2016. The details of the sampled population and sampling methodology of this study have been described previously (18,19,20). Some of the aspects of the sampling procedure should be reiterated. Sampling was conducted in four major cities of Pakistan. These were: Lahore which is the second most populous city of Pakistan, with a high human development index (HDI=0.877); the city of Multan in the center of Pakistan with an HDI=0.718; and two adjacent cities, Rawalpindi and Islamabad, the latter being the capital city of Pakistan, with HDI of 0.871 and 0.875, respectively (21). A grade-wise complete list of schools (i.e., primary and secondary schools) of the selected cities was obtained from Punjab and the Federal Department of Education (Schools). Schools were chosen using simple random sampling from the lists. In each selected school, classes were also selected randomly and all the children who were present on the day of data collection were invited to participate in the study. For this investigation, a sample of 7,921 children, aged 5-14, were recruited from a total of 68 schools of which 28 were Public schools and 40 were Private schools.

After obtaining written consent from the school’s head master and verbal consent from each child’s parents or guardians, data collection activities were performed. All information related to age (years), sex, residential city, and anthropometric measurements including height (cm), weight (kg) and NC (cm) of each child were chronicled in a self-designed questionnaire. Age of each child was confirmed from the school register and physical measurements were taken in a standing position using a standard protocol (20,22). For anthropometric measurements, a stadiometer (Seca model SCA 217, Hamburg, Germany) was used for height and a weighing machine (Westpoint model WF 7009, Karachi, Pakistan) for weight. NC of the children was measured in centimeters using a non-stretchable plastic tape measure. Measurement was made in a horizontal plane, with the participants’ shoulders down and looking straight ahead, at a point just below the thyroid cartilage and perpendicular to the long axis of the neck. This location was chosen, as it is the most easily palpable landmark of the pediatric airway. During the measurement process, attention was paid not to engage the trapezoid muscles of the shoulder and neck. The average of two readings was used for the analysis. All NC measurements were performed by three well-trained data collection teams, supervised by the principal investigator. The BMI of each child was calculated using the standard formula: weight (kg)/height (m^2^). Age-and sex-specific BMI z-scores were obtained by using the LMS method (23). For defining overweight and obesity of a child, World Health Organization 2007 z-scores cut-offs [>+1 standard deviation (SD) i.e. BMI z-score >1 for overweight; and >+2 SD i.e. BMI z-score >2 for obesity] were used. If BMI z-score is <-2, the child will be considered as underweight (24,25).

### Statistical Analysis

The Statistical Package for the Social Sciences (SPSS), version 21.0 was used for all the statistical analyses (IBM Inc., Armonk, NY, USA). For descriptive analysis, means±SD and 95% confidence intervals (CI) were estimated for each sex, based on age in years for each year and age groupings (5-9 and 10-14 years old). Mean differences of NC between two groups were determined using an unpaired t-test. For both sexes, the correlation between NC and other quantitative variables were estimated using Pearson’s correlation. Odds ratios (ORs) were also computed to determine the strength of association. Age-and sex-specific diagnostic ability and cut-off values of NC were calculated with receiver operating characteristic (ROC) curve analysis according to two dependent variables; overweight defined by BMI z-score >1 and obesity defined by BMI z-score >2 (24,25). An NC value with the highest Youden’s index was chosen for best cut-off point. The diagnostic ability of NC to discriminate children with or without overweight and obesity was assessed using area under the curve (AUC). The diagnostic test was considered to be “highly accurate if, 0.65 ≤ AUC ≤ 1.00” and “moderately accurate if, 0.50 ≤ AUC ≤ 0.65” (26,27). The likelihood ratios (positive [LR^P^] and negative [LR^N^]) for NC were also computed for each age and sex as described by Nafiu et al (28). Sex-specific NC cut-off points according to puberty periods were also determined. Boys between 5-11 years and girls between 5-10 years were considered to be in the prepubertal period; boys and girls over 11 and 10 years, respectively were in pubertal period. These age-groupings were chosen as previously described (13).

For this study, exclusion criteria were: (a) children who refused to perform anthropometry (b) children who had goiter or any physical disability and (c) children who were absent at the time of data collection. The study was approved by the Departmental Ethics Committee of Bahauddin Zakariya University, Multan, Pakistan (IRB# SOC/D/2715/19).

## Results

A total of 7921 children, aged 5-14, years were included in the study. The mean BMI and NC were 16.16 Kg/m^2^ and 26.00 cm, respectively. Age-and sex-specific mean (±SD) and 95% CI of each anthropometric measurement are listed in Table 1. For each anthropometric variable, as expected, mean increased with age in both boys and girls. Generally, boys had higher mean values than girls with few exceptions.

Table 2 presents age-and sex-specific mean comparison of NC according to overweight and obesity status. Overweight and obesity prevalence in overall subjects were 16.0% and 3.3%, respectively. Moreover, 1.9% children were underweight (i.e., BMI <-2 SD) in the study. For both genders in different age groups, it was observed that the mean value of NC was higher in subjects that were overweight or obese than in the other subjects. The results were statistically significant at different ages with the exception of 7-year old obese boys.

The correlation coefficients of NC with other anthropometric measurements are displayed in Table 3a. NC had a strong positive correlation with age and all the other anthropometric measures in both genders, as well as in all the subjects studied. Logistic regression analysis confirmed that NC had a statistically significant positive association with overweight and obesity. The crude ORs for overweight and obesity were 1.43 (95% CI: 1.39, 1.46) and 1.42 (95% CI: 1.36, 1.49) and adjusted ORs for overweight and obesity were 1.74 (95% CI: 1.67, 1.80) and 1.76 (95% CI: 1.67, 1.86), respectively (Table 3b).

**Figure 1:**
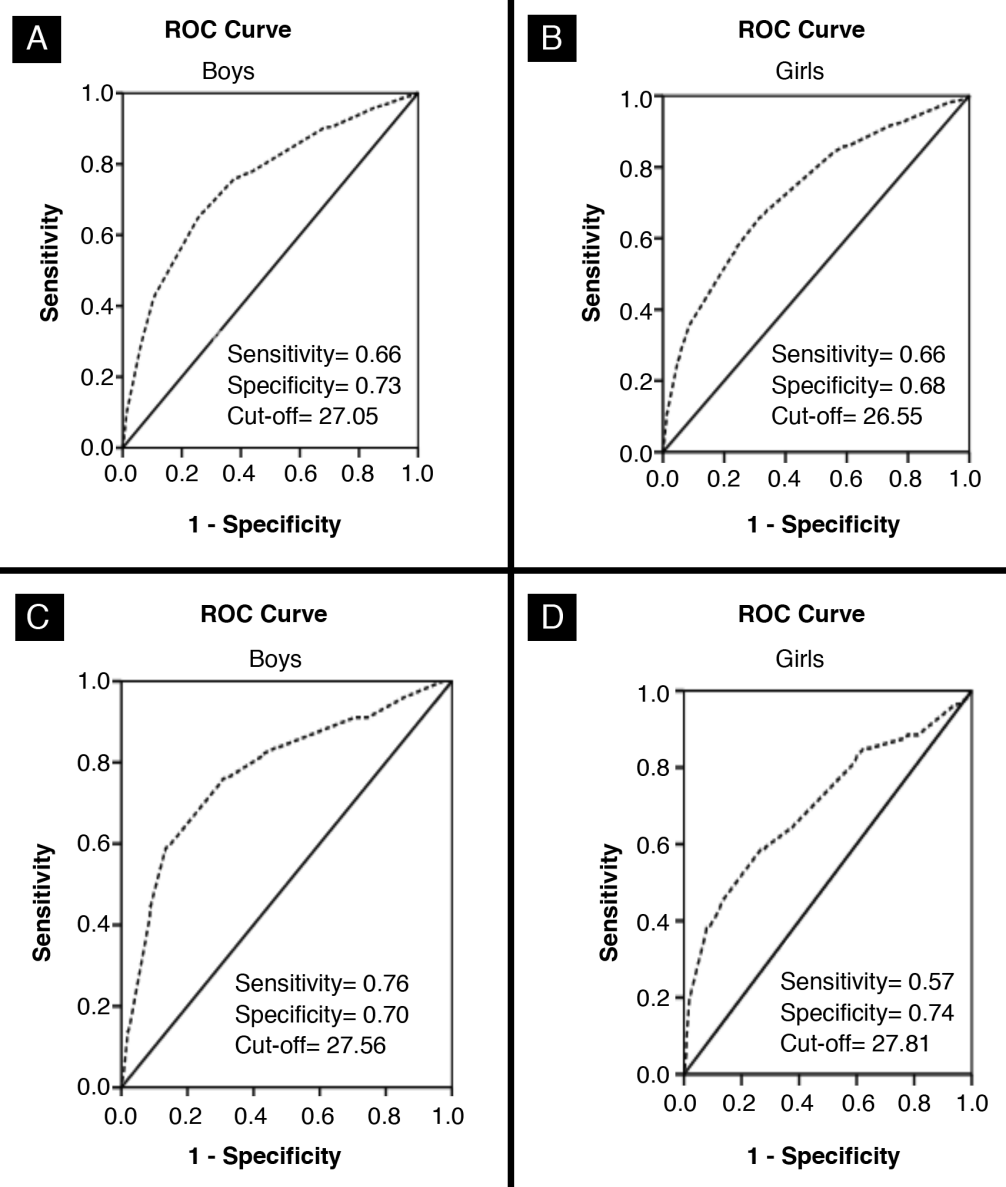
Receiver operating characteristic curve of neck circumference as an indicator of overweight (A+B) and obese (C+D) Pakistani children aged, 5-14 years in both genders ROC: receiver operating characteristic

Table 4 displays the results of AUC for boys and girls of all ages (5-14 years). In all age-groups of both genders, diagnostic performance of NC was ‘highly accurate’ in classifying the individuals to overweight (AUC=0.67 to 0.83) and obesity (AUC=0.66 to 0.97). Diagnostic performance comparison between participants in the prepubertal and pubertal periods showed that the AUC was statistically lower in the prepubertal period. For example, for prepubertal boys the AUC of overweight (0.75) and obesity (0.78) was lower than the AUC values for pubertal overweight (0.78) and obese boys (0.85). The ROC curves accurately define overweight and obesity of the whole cohort regardless of age for both sexes of Pakistani children (see Figure 1).

Based on ROC analysis, sensitivities, specificities, and cut-off values for NC for each age-group, by gender, are presented in Table 5. NC cut-off values for overweight and obesity increased from 25.00 to 30.35 cm for boys and 24.00 to 31.62 cm for girls between 5 and 14 years. In the prepubertal period, NC cut-off values for overweight and obesity were 26.36 and 26.78 cm in boys and 25.27 and 25.02 cm, in girls, respectively. For the pubertal period, these cut-off values were 28.32 and 28.57 cm in boys and 28.70 and 28.82 cm in girls. Considering all the children included in the study, the cut-off points for NC that identified overweight and obesity in boys and girls were 27.05 cm and 27.56 cm for boys and 26.55 cm and 27.81 cm for girls, respectively. The LRs for each cut-off point were also calculated. For example, LRP for a 14-years old boy with NC >30.35 cm indicates that he is 2.64 times more likely to be overweight than a 14-year old boy with an NC value below this cut-off point.

## Discussion

Obesity in children is now considered to be a serious chronic health issue in most populations (29) and its worldwide prevalence is growing (30). Various studies (3,31) have reported increased adverse health outcomes of childhood obesity with both short-term and long-term consequences. Early prevention and treatment of childhood obesity are important priorities for health practitioners and these require accurate diagnostic measures (32). Different practical methods such as BMI, waist circumference (WC), and waist-to-hip ratio are applicable for assessing obesity. However, in circumstances where these methods are not feasible, measurement of NC may be an alternative. NC is a reliable and easy to use index that is generally acceptable to patients and health practitioners (12,13,15). Some studies (12,13) in the pediatric age group have confirmed that NC value measurements could be used as an index of overweight and obesity. In response to these reports, this study was planned to assess the use of NC in Pakistani children using BMI SDS scores to define overweight and obesity.

Validation of NC versus WC and BMI, reported by Hatipoglu et al (13), showed that NC could serve as an easy way to determine overweight and obesity in children with good correlation to cardiovascular risk factors. A study in Greek children, aged 9-13 years, also indicated that NC is associated with cardiovascular risk factors (33). Moreover, the NC measurement was confirmed as a reliable anthropometric index to predict children with cardio-metabolic disease (34).

In the present study it was shown that NC has a good correlation with BMI and other anthropometric characteristics. These findings are consistent with earlier studies (14,35) that reported that NC had a significant positive correlation with age and anthropometric variables in both genders. The NC increased with age in both genders and mean values of NC were higher in overweight and obese children as compared to normal weight subjects. These findings are in accordance with a previous population-based study of Iranian children and adolescents, aged 6-18 years (36). Also consistent with more recent studies (37,38), the present study yields NC in overweight/obese adolescents that are significantly higher than adolescents with normal BMI (p<0.001).

In our study, results for AUC values between 70% and 90% in various age-groups were similar to those found in the Iranian cross-sectional study (36), suggesting that NC could serve to accurately identify children who are overweight or obese. Another Brazilian study, Souza et al (39), has also established NC as an adequate indicator to identify adolescents with high BMI. Similar to two recent studies (40,41), our results also suggest that NC has good diagnostic ability, as indicated by an AUC >0.65, for identifying overweight and obesity in children and adolescents and could be used to screen for excess body weight in routine medical practice. Furthermore, the cut-off point of NC to identify children who are overweight in different age-groups was between 25.00-30.35 cm and 24.00-29.33 cm for boys and girls, respectively. The cut-off points for NC to identify children who are obese in different age-groups was between 25.27-30.35 cm and 25.00-31.62 cm, for boys and girls; respectively. Larger NC cut-offs, between 28.0 to 38.0 cm in boys and 27.0 to 34.5 cm in girls were reported by Hatipoglu et al (13) for a Turkish study for the prediction of overweight and obesity, defined as BMI above the 85th percentile of the BMI reference curve. Similarly, larger cut-off values of NC for the prediction of overweight (defined as BMI between the 85^th^ and 94^th^ centiles for age and sex) or general obesity (defined as obesity as BMI equal to or greater than the sex-specific 95th centile), were also noted in an Iranian population-based study (36). Taheri et al (16) compared the reported NC cut-offs, and associated sensitivity and specificity from different countries and this revealed a notable variation in these values from country to country. Differences in the methods used to define overweight and obesity might partially explain the heterogeneity in the optimal cut-offs among different populations. The variation in sensitivity and specificity of the NC method between studies may be explained due to sample size and age range differences. Furthermore, in our study, BMI-for-age z-scores were calculated by using the LMS method. No other study in the literature calculated BMI-for-age z-scores using this method. Such methodological diversity can also influence these values. The optimal cut-off may vary according to age and additional studies using the same methodology and assessing a wide age range are needed.

Our study has several strengths. Firstly, we have taken a large sample. Secondly, our results using ROC curve analysis are likely to be representative of today’s children and these results are applicable at the national level. Thirdly, there is no similar study to determine the best cut-off points of NC for identification of overweight and obese Pakistani children using a multi-ethnic data set.

Moreover, NC measures were collected by the same researcher, which reduces possible inter-observer biases.

### Study Limitations

The first limitation of this study is that the causality underlying the observed relationships could not be investigated, due to the cross-sectional design. A second limitation is that our study does not cover all age ranges of children and adolescents from birth to 18 years of age. A third limitation is the completely urban and relatively wealthy study population. Findings of the study do not truly cover the rural and relatively poor population of children and adolescents in Pakistan. It should be noted that NC measurements for obesity/overweight screening may be unreliable for individuals with different health problems affecting the neck, such as malignancy or thyroid diseases, cervical spinal disorders, short neck, craniofacial anomalies or neurological conditions or underlying cardiac or pulmonary disease. In spite of the limitations, we believe that the results of this study will contribute new information for knowledge of Public Health.

## Conclusion

NC had good correlation with BMI and also had good diagnostic performance for identifying overweight and obese children. Therefore, NC may be a simple and valuable tool for screening children for weight problems. The results suggested that the Pakistani boys and girls, aged 5-14 years with NC range ≥25.00 to 30.35 cm and ≥24.00 to 31.62 cm, respectively, could be considered to be overweight and obese. As previous epidemiological studies have reported an association between NC and cardiovascular and metabolic risk in obese children and adults, further studies in Pakistani children and young adults should be undertaken to investigate the usefulness of NC as an index of adiposity.

## Figures and Tables

**Table 1:**
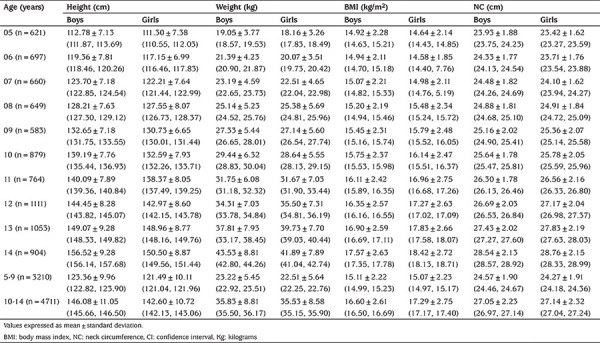
Descriptive statistics (95% confidence interval) for height, weight, body mass index and neck circumference by age

**Table 2:**
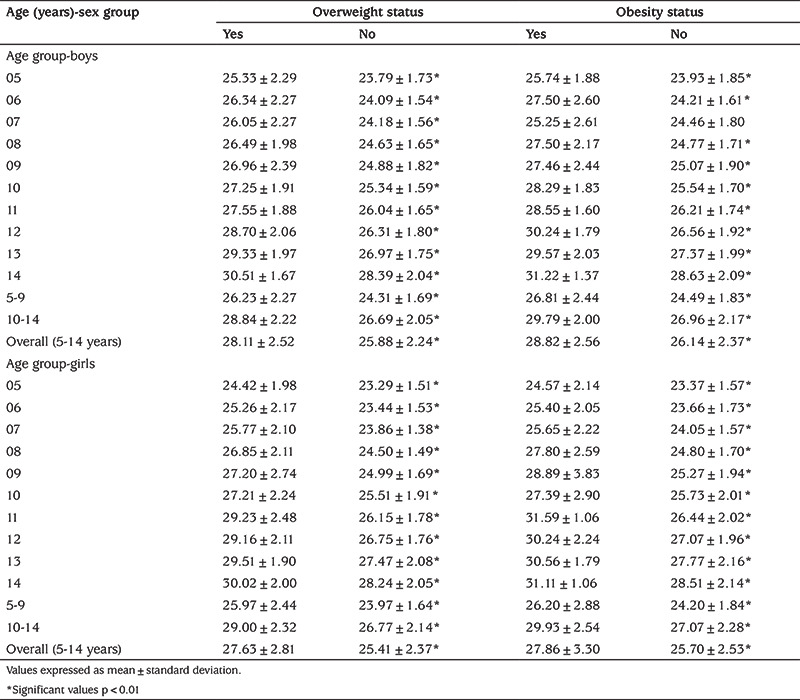
Mean comparison of neck circumference according to overweight and obesity status in children by age and sex

**Table 3a:**
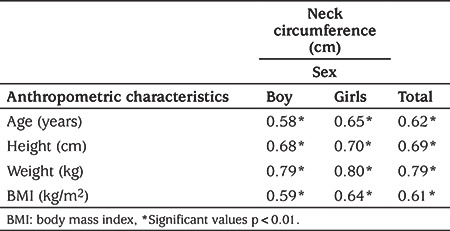
Correlation co-efficient between neck circumference and other anthropometric characteristics in children

**Table 3b:**
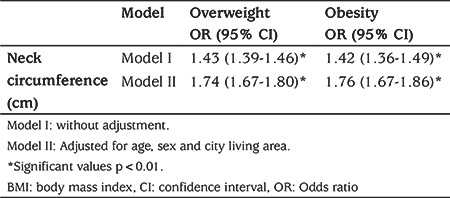
Association of neck circumference with overweight (i.e. body mass index z-score >1) and obesity (i.e. body mass index z-score >2) in a logistic regression model

**Table 4:**
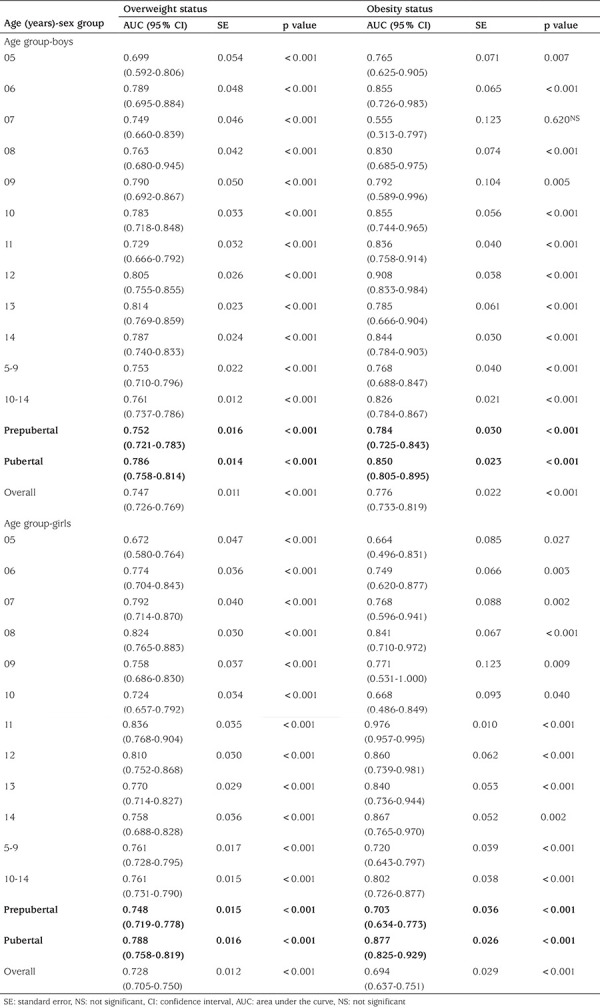
Area under the curve for detection of overweight and obesity based on the neck circumference in children by age and sex

**Table 5:**
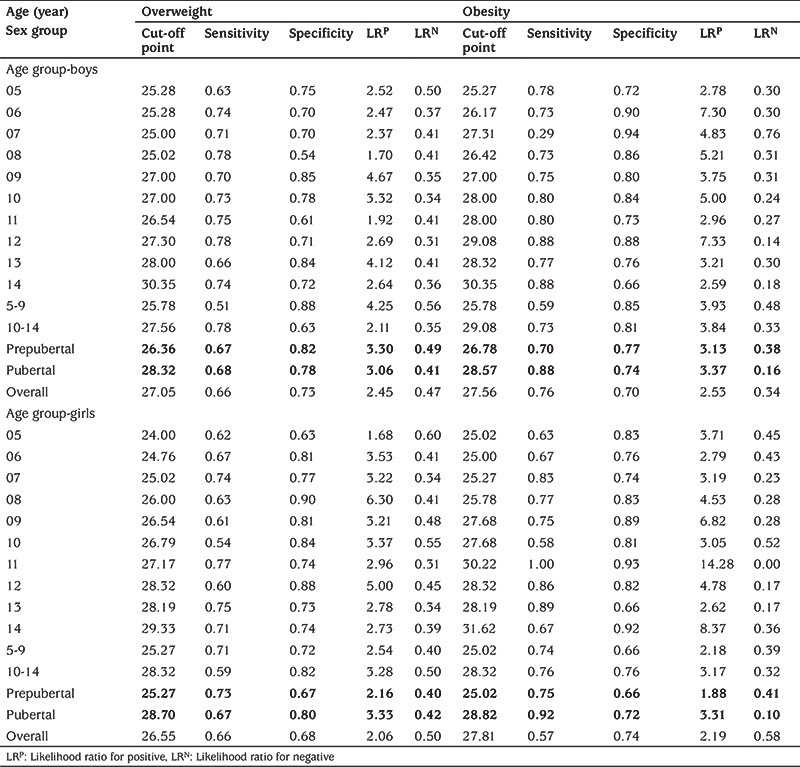
Cut-off point, sensitivity and specificity of neck circumference for detecting overweight and obesity in children by sex and age groups
